# Assessing the Evidence for Asymmetrical Switch Costs and Reversed Language Dominance Effects – A Meta-Analysis

**DOI:** 10.5334/joc.186

**Published:** 2021-09-13

**Authors:** Miriam Gade, Mathieu Declerck, Andrea M. Philipp, Alodie Rey-Mermet, Iring Koch

**Affiliations:** 1 Catholic University of Eichstätt-Ingolstadt, Department of Psychology, General Psychology; 2 Medical School Berlin, Department of Sciences, Berlin, Germany; 3 Vrije Universiteit Brussel, Department of Linguistics and Literary Studies, Brussels, Belgium; 4 RWTH Aachen University, Department of Psychology, Aachen, Germany; 5 UniDistance Suisse, Brig, Switzerland

**Keywords:** bilingualism, language control, cognitive control, language production

## Abstract

Two seemingly counterintuitive phenomena – asymmetrical language switch costs and the reversed language dominance effect – prove to be particularly controversial in the literature on language control. Asymmetrical language switch costs refer to the larger costs for switching into the dominant language compared to switching into the less dominant language, both relative to staying in either one language. The reversed language dominance effect refers to longer reaction times when in the more dominant of the two languages in situations that require frequent language switching (i.e., mixed-language blocks). The asymmetrical language switch costs are commonly taken as an index for processes of transient, reactive inhibitory language control, whereas the reversed language dominance effect is taken as an index for sustained, proactive inhibitory language control. In the present meta-analysis, we set out to establish the empirical evidence for these two phenomena using a Bayesian linear mixed effects modelling approach. Despite the observation of both phenomena in some studies, our results suggest that overall, there is little evidence for the generality and robustness of these two effects, and this holds true even when conditions – such as language proficiency and preparation time manipulations – were included as moderators of these phenomena. We conclude that asymmetrical switch costs and the reversed language dominance effect are important for theory development, but their utility for theory testing is limited due to their lack of robustness and the absence of confirmed moderatory variables.

## Introduction

Imagine a German family being on vacation in France and going to a bakery. One of the parents collects the orders of the non-French speaking family members in German and then addresses the salesperson in French, transmitting all wishes and taking into account last minute changes of the preferred items by the kids in German. To do so successfully, the parent has to switch between German and French repeatedly in both comprehension (i.e., taking the orders by the family members and understanding the questions by the French salesperson) as well as in production (i.e., ensuring to order the right items by asking the kids and ordering them by engaging in conversation with the salesperson). This is a poignant example of language control, which is the process that makes it more likely that words from the target language will be selected while the other language is active. An abundance of research has shown that language control is almost always necessary during language processing of bilinguals. This is because next to the target language, the non-target language is generally activated in parallel, and sometimes selected (e.g., Declerck, Lemhöfer, et al., 2017; Gollan et al., 2011), even when bilinguals are in a single language context (e.g., Costa et al., 2000; Lee et al., 2019; Thierry & Wu, 2007). However, which effects of language control are likely to be observed and their link to underlying mechanisms is yet to be assessed, which motivated the present study.

From a cognitive psychologist’s viewpoint, how the language selection process takes place and which difficulties participants encounter when selecting one language over the other has been a topic of much research in the last two decennia, most of which relied on the language switching paradigm in its various variants (for recent reviews Blanco-Elorrieta & Pylkkänen, 2018; Calabria et al., 2018; Declerck & Philipp, 2015). In the current study, we investigate the selection of languages by evaluating the evidence in favour of two phenomena repeatedly reported when switching between languages: the asymmetry in language switch costs and the reversed language dominance effect. Both phenomena occur when participants are asked to switch between languages of different dominance levels in mixed-language blocks. Mixed-language blocks are blocks in which participants are asked to switch between both languages on a trial-by-trial basis. Whereas the asymmetry in language switch costs refers to larger switch costs in the dominant language compared to the nondominant language, the reversed language dominance effect refers to generally worse performance in the dominant language than the less dominant language in such mixed-language blocks. Thus, investigating both effects requires at least a 2 × 2 within-subject design. In this design, the first independent variable is typically a language transition factor with two levels, language switch vs. language repetition, and the second independent variable is a language dominance factor with two levels, dominant language vs. less dominant language. With such a design, the switch costs – that is, the worse performance when switching languages compared to repeating the language – are indicated by a main effect of language transition. Critically, the asymmetry in language switch costs is then indicated by an interaction between language transition and language dominance, and the (reversed) language dominance effect is indicated by a main effect of language dominance.

At the theoretical level, both phenomena – that is, the asymmetry in language switch costs and the reversed language dominance effect – have been argued to reflect different control modes (Declerck, 2020). That is, asymmetrical language switch costs have been assumed to indicate reactive or transient language control, whereas the reverse language dominance effect has been assumed to reflect proactive or sustained language control. Yet, for both modes, language control is assumed to be primarily achieved via suppression of one language (i.e., inhibitory control; see Green, 1998).

However, despite the theoretical relevance of the asymmetrical switch costs and the reversed language dominance effect, the empirical results are mixed. Whereas several studies report (both or one of) these effects (i.e., Bonfieni et al., 2019; Christoffels et al., 2007; Philipp et al., 2007; Meuter & Allport, 1999), many studies did not observe these effects even under conditions in which they would have been expected (Costa et al. 2006, Experiment 1 and 2; Ma et al., 2016). Given this apparent lack of empirical robustness across and within studies, we aim at establishing boundary conditions under which those phenomena are more likely to be observed. To this end, we reanalysed the published literature to establish the presence of asymmetrical switch costs and a reversed language dominance effect across the range of available language-switching studies using a meta-analytic approach.

## Switch Costs and their Asymmetry

Language control is commonly investigated using the language-switching paradigm. In a language-switching experiment, bilinguals usually either have to name digits or pictures in one of their languages, as indicated either by an explicit language cue (e.g., a geometric shape or colour presented before or simultaneously with the imperative stimulus; e.g., Meuter & Allport, 1999), a pre-instructed sequence (i.e., AABB, whereby A and B stand for the two languages, respectively; e.g., Declerck et al., 2013), or based on voluntary language selection (e.g., Gollan & Ferreira, 2009). Compared to a repetition of the language, performance suffers, in both reaction time (RT) and error rate (ER), when the language changes across trials, thus representing switch costs (see Declerck & Philipp, 2015, for a review).

It has repeatedly been reported that switch costs are influenced by the dominance of the language participants switch to, in that switching to the more dominant language incurs a larger cost than switching to the less dominant language. The observation of asymmetrical switch costs is typically explained with inhibition (Green, 1998; Meuter & Allport, 1999; for alternative approaches, see Finkbeiner et al., 2006; Philipp et al., 2007; Verhoef et al., 2009): According to this account, processing of a specific language on trial n results in the inhibition of the non-target language. This inhibition persists across the subsequent trial (i.e., trial n + 1). In a switch trial – that is, when switching from one language to another language – the language that was inhibited in the previous trial should be reactivated, thus resulting in a language switch cost. However, the dominance of the language further biases this process. That is, when the language to be used is the less dominant one, more inhibition is necessary on trial n to suppress the dominant but irrelevant language. Thus, in the subsequent trial, it is more difficult to reactivate the dominant and now relevant language, thus resulting in larger switch costs when switching to the dominant language.

Yet, the notion of more inhibition being implemented towards the more dominant language has been challenged on several fronts. For example, several studies have observed *symmetric* switch costs for highly proficient bilinguals that switched between the dominant language and a third language, whereby the third language is clearly less proficient (e.g., Costa & Santesteban, 2004; Costa et al., 2006). According to the inhibition account laid out above, this should have resulted in larger switch costs for any of the two more dominant languages than for the third, least dominant language. Costa and colleagues explained this pattern by assuming that highly proficient bilinguals do not necessarily rely on inhibitory control but rely on a different language control process in which they can only select words from their target language, and this different language control processes abolishes the asymmetry. However, symmetric switch costs have also been observed with second language learners who are clearly dominant in their native language, that is, under conditions for which the asymmetry should be observed if language dominance is the relevant modulatory variable (e.g., Declerck et al., 2012; Finkbeiner et al., 2006; Heikoop et al., 2016; Mosca & de Bot, 2017; Peeters & Dijkstra, 2018).

In addition to the findings showing no asymmetrical language switch costs for which switching to the dominant language is more costly, there are also reports of an inversed asymmetry. That is, switch costs were larger for the less dominant language than for the dominant language (Bonfieni et al., 2019; Declerck, Stephan et al., 2015; Zheng et al., 2020; see also Liu et al., 2019). Declerck, Stephan et al. (2015) speculated that this might be due to a reversal of the language dominance in mixed language blocks (i.e., better overall performance in the less dominant than the dominant language), resulting in more inhibition of the less dominant language during dominant language trials, and thus a larger cost to overcome this inhibition when switching back to the less dominant language. Although the pattern of results combining a reversed language dominance and a reversed asymmetrical switch costs was observed in some studies (Bonfieni et al., 2019; Declerck, Stephan et al., 2015; Zheng et al., 2020), there are also studies reporting a reversed language dominance pattern without a (reversed) asymmetrical switch costs (e.g., Christoffels et al., 2007; Heikoop et al., 2016; Verhoef et al., 2009).

One possible reason for this mixed evidence might be that in most studies, language dominance was not assessed using objective measures. Rather, it was assessed using self-report measures (for a discussion on bilingual language assessment, see de Bruin, 2019) or the age-of-acquisition of the less dominant language (assuming the dominant language was learnt earlier, unless in case of immersion where the latter learnt societal language might affect language dominance because of education; see Segal et al., 2019). Thus, the absence of objective measures across studies complicates the comparability of language dominance across the studies, and thus the conclusiveness of the observed pattern of asymmetrical switch costs.

Taken together, these findings question the robustness of asymmetrical switch costs and thus potentially call for specification of the inhibitory account that is predominantly used to explain these asymmetrical switch costs. So far, the field has attempted to answer the question of whether language selection goes along with asymmetrical switch costs with narrative reviews (e.g., Bobb & Wodniecka, 2013; Declerck & Philipp, 2015). Therefore, the main aim of this work is to complement those narrative reviews with a more recent, comprehensive quantitative meta-analysis of published studies to examine whether there is evidence in favour of asymmetrical switch costs.

## Language Dominance Effects

In addition to investigating the asymmetry in language switch costs, we were also interested in the reversed language dominance effect in mixed-language blocks. In single-language blocks, naming responses in the dominant language are usually faster than in the less dominant (or even least dominant) language. This RT benefit is assumed to arise because of an activation advantage for the more dominant language. If activation levels differ among languages, then the dominant language should always lead to a benefit (i.e., a language dominance effect). However, such an effect is not always observed. Specifically, some studies found a reversal of the language dominance pattern in mixed-language blocks (e.g., Christoffels et al., 2007; Costa & Santesteban, 2004; Gollan & Ferreira, 2009; Heikoop et al., 2016; Verhoef et al., 2009; for a review see Declerck, 2020).

This *reversed* language dominance effect has been explained by assuming that overall performance in mixed-language blocks is optimal when both languages have a similar activation level (for evidence along these lines, see Declerck et al., 2020). To achieve this, the more dominant language is assumed to be proactively inhibited, resulting in a more sustained, general slowing of performance in the dominant language. However, participants might not be able to recruit the exact amount of sustained inhibition necessary to reach a comparable activation level of both languages. Thus, the dominant language might be inhibited too strongly, so that even a reversal of language dominance might occur (see Declerck et al., 2020).

In previous studies, a diverse pattern of results was found. A first group of studies report the usually predicted advantage of the dominant language over the less dominant language even in mixed-language blocks (e.g., Ma et al., 2016; Wang et al., 2009). A second group of studies found no overall language effect (Calabria et al., 2015; Prior & Gollan, 2011), whereas a third group of studies observed a reversed language dominance effect (e.g., Christoffels et al., 2007; Costa & Santesteban, 2004; Gollan & Ferreira, 2009; Heikoop et al., 2016; Verhoef et al., 2009; Zheng et al., 2018). Consequently, the robustness of the reversed language dominance effect in mixed-language conditions has been questioned (e.g., Declerck, 2020).

Exploring a (reversed) language dominance effect becomes even more challenging as it has been argued that dominance effects are not stable throughout one’s lifetime. It is assumed that biographical changes (e.g., increasing formal education, changes in the workplace as well as migration experience) can change the pattern of language dominance (Anderson et al., 2020; Marian & Hayakawa, 2020). Therefore, some researchers argue that it is difficult to give a veridical impression of current language dominance and ask for a more situation-based assessment (Luk, 2015; Marian & Hayakawa, 2020). Another issue refers to the often-used self-assessments of language dominance, for which it has been argued that those are prone to biases (Tomoschuk et al., 2019). Whereas most studies report self-assessments, studies using objective tests such as the LexTale (Lemhöfer & Broersma, 2012) or MINT (Gollan et al., 2012), which are assumed to provide a more precise measure of actual language capabilities, are rare, and researchers have only recently included those in the assessment of participants.

For the present meta-analysis, we decided to use the self-assessed values if no other measures were available in the studies forming the data base. To be more precise, we performed an auxiliary analysis based on language proficiency as indicated by the authors of the individual studies. From these language proficiency scores we computed a language dominance ratio dividing the (self-assessed) proficiency of the less dominant language by the self-assessed proficiency of the dominant language and entered this quotient as a continuous variable to the analysis.

## The Present Study

In this study, we assessed the evidence in favour of both asymmetrical language switch costs and the reversed language dominance effect when switching between languages of (relative) different dominance. That is, we asked whether the specific directionalities (i.e., larger switch costs when switching to the more dominant language and faster reactions in the less dominant language in mixed language blocks) receive empirical support given the results reported overall in the literature.

Based on the theoretical framing in terms of inhibitory control and the diversity of the previous empirical findings, three scenarios can be expected. First, because the reversed dominance effect has been linked to sustained, proactive language control adjusting the overall activation level of the two languages and the asymmetrical switch costs have been proposed to reflect transient, reactive language control arising from the deployment of inhibition (Declerck, 2020; Green, 1998), it is possible that both processes might be interwoven. That is, strong sustained language control reduces the need for transient reactive control. In this case, evidence for reversed language dominance should be observed but no evidence for asymmetrical switch costs should be found. Second, it is also conceivable that sustained and transient control of languages operate on different time scales and no mutual influence is observed. In this case, positive (or null) evidence for both the reversed language dominance effect and the existence of asymmetrical switch costs should be observed. Third, it is possible that both effects are affected not only by language dominance but also by other factors, such as the language dominance ratio, type of language switching paradigm, or the preparation time (i.e., the time between the cue indicating which language to use and the target stimulus). In this case, both effects might be observed but should additionally be influenced by (by the same or different) moderating variables. Overall, we think that this quantitative assessment will help to advance the field of bilingual language control by allowing to decide among the scenarios outlined above.

## Method

### Samples of study

We ran a systematic literature review on available studies. To this end, we searched the databases PubMed, Google Scholar and PsycInfo as well as the reference lists of published studies. Keywords to look for studies were “language switching”, “voluntary switching”, “bilingualism”, “bilingual flexibility”. We limited our search to already published or accepted work by the end of the search period (April 1^st^, 2021). We are thankful to the authors we contacted who provided relevant, missing information allowing us to perform this study. In sum, this meta-analysis was based on data we extracted from 73 studies that met our inclusion and exclusion criteria (see below). Those studies forming the data basis of the current work can be found in ***Table 1*** with their reference, type of paradigm, languages used as well as information on number of random effects, language dominance ratio, and the assessment of timing manipulations. Raw data underlying the analyses as well as analyses scripts can be found at *https://osf.io/ukjq4*.

**Table 1:** Studies included in the present meta-analysis.


NUMBER OF STUDY	REFERENCE	PARADIGM	NUMBER OF RANDOM EFFECTS	LANGUAGES ASSESSED^1^	RATIO PROFICIENCY LESS DOMINANT/DOMINANT (%)^2^	TIMING

1	Blanco-Elorrieta, E., & Pylkkänen, L. (2017). Bilingual language switching in the laboratory versus in the wild: The spatiotemporal dynamics of adaptive language control. *Journal of Neuroscience, 37*, 9022–9036. *https://doi.org/10.1523/JNEUROSCI.0553-17*.	cued	3	**Arabic**/English	84.5	CTI 300 ms

2	Bonfieni, M., Branigan, H. P., Pickering, M. J., & Sorace, A. (2019). Language experience modulates bilingual language control: The effect of proficiency, age of acquisition, and exposure on language switching. *Acta Psychologica, 19*3, 160–170. *https://doi.org/10.1016/j.actpsy.2018.11.004*	cued	2	**Italian**/English **Italian/**Sardinian	83.94 95.42	CTI 0 ms

3	Calabria, M., Branzi, F. M., Marne, P., Hernández, M., & Costa, A. (2015). Age-related effects over bilingual language control and executive control. *Bilingualism: Language and Cognition, 18*, 65–78. *https://doi.org/10.1017/S1366728913000138*	cued	2	**Catalan/**Spanish	97.50 52.50	CTI 1000 ms

4	Calabria, M., Hernández, M., Branzi, F. M., & Costa, A. (2012). Qualitative differences between bilingual language control and executive control: Evidence from task-switching. *Frontiers in Psychology, 2, https://doi.org/10.3389/fpsyg.2011.00399*	cued	3	**Catalan/**Spanish	97.50 100.00 92.50	CTI 1000 ms

5	Campbell, J. I. D. (2005). Asymmetrical language switching costs in Chinese–English bilinguals’ number naming and simple arithmetic. *Bilingualism: Language and Cognition, 8*, 85–91. *https://doi.org/10.1017/S136672890400207X*	cued	6	**Chinese**/English	60.00 61.67 61.67 61.67 61.67 61.67	CTI 1000 ms

6	Christoffels, I. K., Fink, C., & Schiller, N. O. (2007). Bilingual language control: An event-related brain potential study. *Brain Research, 1147*, 192–208. *https://doi.org/10.1016/j.brainres.2007.01.137*	cued	2	**German**/Dutch	63.64	not given

7	Contreras-Saavedra, C. E., Koch, I., Schuch, S., & Philipp, A. M. (2021). The reliability of language-switch costs in bilingual one- and two-digit number naming. *International Journal of Bilingualism, 25*, 272–285. *https://doi.org/10.1177/1367006920951873*	cued	3	**German/Spanish/**English	77.65^+^	CTI 100ms

8	Costa, A., & Santesteban, M. (2004). Lexical access in bilingual speech production: Evidence from language switching in highly proficient bilinguals and L2 learners. *Journal of Memory and Language, 50*, 491–511. *https://doi.org/10.1016/j.jml.2004.02.002*	cued	7	**Korean**/Spanish **Spanish/**Catalan **Spanish**/English	63.64 68.99 57.50 92.41 94.18 70.13 94.46	CTI 2000 ms SOA^§^ 500/800 ms

9	Costa, A., Santesteban, M. l, & Ivanova, I. (2006). How do highly proficient bilinguals control their lexicalization process? Inhibitory and language-specific selection mechanisms are both functional. *Journal of Experimental Psychology: Learning, Memory, and Cognition, 32*, 1057–1074. *https://doi.org/10.1037/0278-7393.32.5.1057*	cued	6	**Spanish**/Basque **Spanish**/English **Catalan/**English **Spanish/**new language	94.74 92.50 58.33 53.33 92.50 47.50	CTI 2000 ms

10	de Bruin, A., Samuel, A. G., & Duñabeitia, J. A. (2018). Voluntary language switching: When and why do bilinguals switch between their languages? *Journal of Memory and Language, 103*, 28–43. *https://doi.org/10.1016/j.jml.2018.07.005*	cued & voluntary	3	**Basque/**Spanish	89.25 92.63 92.63	CTI 500 ms

11	Declerck, M., & Philipp, A. M. (2015). The unusual suspect: Influence of phonological overlap on language control. *Bilingualism: Language and Cognition, 18*, 726–736. *https://doi.org/10.1017/S1366728914000236*	cued	2	**German/**English	70.00 84.29	CTI 500 ms

12	Declerck, M., Stephan, D. N., Koch, I., & Philipp, A. M. (2015). The other modality: Auditory stimuli in language switching. *Journal of Cognitive Psychology, 27*, 685–691. *https://doi.org/10.1080/20445911.2015.1026265*	alternating runs	2	**German/**English	72.86 72.86	not applicable

13	Declerck, M., Koch, I., & Philipp, A. M. (2012). Digits vs. pictures: The influence of stimulus type on language switching. *Bilingualism: Language and Cognition, 15*, 896–904. *https://doi.org/10.1017/S1366728912000193*	cued	4	**German**/English	70.00 70.00 70.00 70.00	CTI 1000 ms

14	Declerck, M., Philipp, A. M., & Koch, I. (2013). Bilingual control: Sequential memory in language switching. *Journal of Experimental Psychology: Learning, Memory, and Cognition, 39*, 1793–1806. *https://doi.org/10.1037/a0033094*	alternating runs	8	**German**/English	67.14 67.14 75.71 75.71 71.43 71.43 67.14 67.14 74.29	not applicable

15	Declerck, M., Koch, I., & Philipp, A. M. (2015). The minimum requirements of language control: Evidence from sequential predictability effects in language switching. *Journal of Experimental Psychology: Learning, Memory, and Cognition, 41*, 377–394. *https://doi.org/10.1037/xlm0000021*	alternating runs/cued	10	**German**/English	74.29 64.29 64.29 64.29 68.57 74.29 74.29 68.57 72.86 72.86	CTI 0 ms

16	Declerck, M., Thoma, A. M., Koch, I., & Philipp, A. M. (2015). Highly proficient bilinguals implement inhibition: Evidence from n-2 language repetition costs. *Journal of Experimental Psychology: Learning, Memory, and Cognition, 41*, 1911–1916. *https://doi.org/10.1037/xlm0000138*	cued, switching between two languages included	0	**German**/English	No self-rated proficiency scores	CTI 100 ms

17	Declerck, M., Ivanova, I., Grainger, J., & Duñabeitia, J. A. (2020). Are similar control processes implemented during single and dual language production? Evidence from switching between speech registers and languages. *Bilingualism: Language and Cognition, 23*, 694–701. *https://doi.org/10.1017/S1366728919000695*	cued	3	**French**/English	83.52^+^ 76.57	Exp 2: CTI 0/800ms

18	Gollan, T. H., & Ferreira, V. S. (2009). Should I stay or should I switch? A cost–benefit analysis of voluntary language switching in young and aging bilinguals. *Journal of Experimental Psychology: Learning, Memory, and Cognition, 35*, 640–665. *https://doi.org/10.1037/a0014981*	voluntary	3	**English/**Spanish	83.82 83.58 81.36	not applicable

19	Gollan, T. H., Kleinman, D., & Wierenga, C. E. (2014). What’s easier: Doing what you want, or being told what to do? Cued versus voluntary language and task switching. *Journal of Experimental Psychology. General, 143*, 2167–2195. *https://doi.org/10.1037/a0038006*	cued & voluntary	4	**Spanish**/English	92.31 98.39 92.31 98.39	CTI 250 ms

20	Graham, B., & Lavric, A. (2021). Preparing to switch languages versus preparing to switch tasks: Which is more effective? *Journal of Experimental Psychology: General*, No Pagination Specified. *https://doi.org/10.1037/xge0001027*	cued	3	**German/French/Spanish**/English	90.83	CTI 50/ 800/ 1175 ms

21	Gross, M., & Kaushanskaya, M. (2015). Voluntary language switching in English–Spanish bilingual children. *Journal of Cognitive Psychology, 27*, 992–1013. *https://doi.org/10.1080/20445911.2015.1074242*	voluntary	0	**Spanish/**English	No self-rated proficiency scores	not applicable

22	Gullifer, J. W., Kroll, J. F., & Dussias, P. E. (2013). When language switching has no apparent cost: Lexical access in sentence context. *Frontiers in Psychology, 4*, 278.^$^	alternating runs	2	**Spanish**/English	94.43 94.43	not applicable

23	Grunden, N., Piazza, G., García-Sánchez, C., & Calabria, M. (2020). Voluntary Language Switching in the Context of Bilingual Aphasia. *Behavioral Sciences, 10*, 141.	voluntary	4	**Spanish/Catalan**	100	not applicable

24	Jevtović, M., Duñabeitia, J. A., & Bruin, A. de. (2019). How do bilinguals switch between languages in different interactional contexts? A comparison between voluntary and mandatory language switching. *Bilingualism: Language and Cognition, 23*, 401–413. *https://doi.org/10.1017/S1366728919000191*	voluntary	2	**Spanish/Basque**	93.60 93.60	not applicable

25	Jylkkä, J., Lehtonen, M., Lindholm, F., Kuusakoski, A., & Laine, M. (2018). The relationship between general executive functions and bilingual switching and monitoring in language production. *Bilingualism: Language and Cognition, 21*, 505–522. *https://doi.org/10.1017/S1366728917000104*	cued	0	**Finnish**/English	84.29	CTI 0 ms

26	Kang, C., Ma, F., & Guo, T. (2018). The plasticity of lexical selection mechanism in word production: ERP evidence from short-term language switching training in unbalanced Chinese–English bilinguals. *Bilingualism: Language and Cognition, 21*, 296–313. *https://doi.org/10.1017/S1366728917000037*	cued	2	**Chinese**/English	63.29 69.60	CTI 800 ms

27	Khateb, A., Shamshoum, R., & Prior, A. (2017). Modulation of language switching by cue timing: Implications for models of bilingual language control. *Journal of Experimental Psychology: Learning, Memory, and Cognition, 43*, 1239–1253. *https://doi.org/10.1037/xlm0000382*	cued	5	**Arabic**/Hebrew	90.00 90.00 90.00 90.00 90.00	CTI 0/300/900 ms TCI 300/900 ms

28	Kirk, N. W., Kempe, V., Scott-Brown, K. C., Philipp, A., & Declerck, M. (2018). Can monolinguals be like bilinguals? Evidence from dialect switching. *Cognition, 170*, 164–178. *https://doi.org/10.1016/j.cognition.2017.10.001*	cued	8	**German**/German Dialect **English**/English Dialect	90.00 90.00 95.57 90.14 95.57 95.57 62.29 62.29	CTI 0 ms

29	Kleinman, D., & Gollan, T. H. (2016). Speaking two languages for the price of one: Bypassing language control mechanisms via accessibility-driven switches. *Psychological Science, 27*, 700–714. *https://doi.org/10.1177/0956797616634633#*	cued	1	**Spanish/**English	90.91	CTI 250 ms

30	Kleinman, D., & Gollan, T. H. (2018). Inhibition accumulates over time at multiple processing levels in bilingual language control. *Cognition, 173*, 115–132. *https://doi.org/10.1016/j.cognition.2018.01.009*	cued, 50% switch condition only taken into account	1	**Spanish**/English	74.74	CTI 250 ms

31	Kubota, M., Chevalier, N., & Sorace, A. (2019). How bilingual experience and executive control influence development in language control among bilingual children. *Developmental Science, e12865*. *https://doi.org/10.1111/desc.12865*	cued	2	**Japanese**/English	No self-rated proficiency scores	CTI 0 ms

32	Lavric, A., Clapp, A., East, A., Elchlepp, H., & Monsell, S. (2019). Is preparing for a language switch like preparing for a task switch? *Journal of Experimental Psychology: Learning, Memory, and Cognition, 45*, 1224–1233. *https://doi.org/10.1037/xlm0000636*	cued	4	**German**/English	90.00 90.00 90.00 90.00	CTI 100/1500 ms

33	Li, S., Botezatu, M. R., Zhang, M., & Guo, T. (2021). Different inhibitory control components predict different levels of language control in bilinguals. *Memory & Cognition*. *https://doi.org/10.3758/s13421-020-01131-4*	cued	2	**Chinese/**English	63.25 67.18	CTI 0 ms

34	Li, C., & Gollan, T. H. (2018). Cognates Facilitate Switches and then Confusion: Contrasting Effects of Cascade versus Feedback on Language Selection. *Journal of Experimental Psychology: Learning, Memory, and Cognition, 44*, 974–991. *https://doi.org/10.1037/xlm0000497*	cued	6	**English/**Spanish	76.55 72.64 68.89	CTI 0 ms

35	Li, C., & Gollan, T. H. (2021). What cognates reveal about default language selection in bilingual sentence production. *Journal of Memory and Language, 118*, 104214. *https://doi.org/10.1016/j.jml.2020.104214*	cued	6	**Spanish/**English	80.72^†^ 78.25 76.25	CTI 0 ms

36	Liu, H., Zhang, M., Pérez, A., Xie, N., Li, B., & Liu, Q. (2019). Role of language control during interbrain phase synchronization of cross-language communication. *Neuropsychologia, 131*, 316–324. *https://doi.org/10.1016/j.neuropsychologia.2019.05.014*	cued	4	**Chinese/**English	62.30	CTI 250 ms

37	Liu, C., Jiao, L., Wang, Z., Wang, M., Wang, R., & Wu, Y. J. (2019). Symmetries of bilingual language switch costs in conflicting versus non-conflicting contexts. *Bilingualism: Language and Cognition, 22*, 624–636. *https://doi.org/10.1017/S1366728918000494*	cued	2	**Chinese/**English	55.72 59.73	CTI 0 ms

38	Liu, C., Timmer, K., Jiao, L., Yuan, Y., & Wang, R. (2019). The influence of contextual faces on bilingual language control. Quarterly Journal of Experimental Psychology, 72, 2313–2327. *https://doi.org/10.1177/1747021819836713*	cued	6	**Chinese/**English	55.59 55.59 55.59 57.65 57.65 57.65	CTI 1000 ms

39	Liu, H., Kong, C., de Bruin, A., Wu, J., & He, Y. (2020). Interactive influence of self and other language behaviors: Evidence from switching between bilingual production and comprehension. *Human Brain Mapping, 41*, 3720–3736.	cued	2	**Chinese/**English	65.39	CTI 0 ms

40	Liu, H., Tong, J., de Bruin, A., Li, W., He, Y., & Li, B. (2020). Is inhibition involved in voluntary language switching? Evidence from transcranial direct current stimulation over the right dorsolateral prefrontal cortex. *International Journal of Psychophysiology, 147*, 184–192. *https://doi.org/10.1016/j.ijpsycho.2019.12.002*	voluntary	3	**Chinese/**English	76.77	not applicable

41	Ma, F., Li, S., & Guo, T. (2016). Reactive and proactive control in bilingual word production: An investigation of influential factors. *Journal of Memory and Language, 86*, 35–59. *https://doi.org/10.1016/j.jml.2015.08.004*	cued	9	**Chinese/**English	73.63 73.63 73.63 68.66 68.66 68.66 73.56 73.56 73.56	CTI 0/500/800 ms TCI 200/500/800 ms RCI 500/800/1500 ms

42	Macizo, P., Bajo, T., & Paolieri, D. (2012). Language switching and language competition. *Second Language Research, 28*, 131–149. *https://doi.org/10.1177/0267658311434893*	reading aloud	1	**Spanish**/English	85.08	not applicable

43	Martin, C. D., Strijkers, K., Santesteban, M., Escera, C., Hartsuiker, R. J., & Costa, A. (2013). The impact of early bilingualism on controlling a language learned late: An ERP study. *Frontiers in Psychology, 4*. *https://doi.org/10.3389/fpsyg.2013.00815*	cued	3	**Spanish**/Catalan/**Spanish**/English	55.00 55.00 97.50	CTI 2000 ms

44	Massa, E., Köpke, B., & El Yagoubi, R. (2020). Age-related effect on language control and executive control in bilingual and monolingual speakers: Behavioral and electrophysiological evidence. *Neuropsychologia, 138*, 107336. *https://doi.org/10.1016/j.neuropsychologia.2020.107336*	?	2	**French**/Italian	62.07 61.67	not applicable

45	Mofrad, F. T., Jahn, A., & Schiller, N. O. (2020). Dual Function of Primary Somatosensory Cortex in Cognitive Control of Language: Evidence from Resting State fMRI. *Neuroscience, 446*, 59–68. *https://doi.org/10.1016/j.neuroscience.2020.08.032*	cued	0	**Dutch/**English	No self-rated proficiency scores	CTI 750 ms

46	Mosca, M., & Clahsen, H. (2016). Examining language switching in bilinguals: The role of preparation time. *Bilingualism: Language and* Cognition, 19, 415–424. *https://doi.org/10.1017/S1366728915000693*	cued	2	**German**/English	75.4	CTI 0/800 ms

47	Mosca, M., & de Bot, K. (2017). Bilingual language switching: Production vs. recognition. *Frontiers in Psychology, 8*. *https://doi.org/10.3389/fpsyg.2017.00934*	cued	0	**Dutch**/English	No self-rated proficiency scores	CTI 0 ms

48	Olson, D. J. (2016). The gradient effect of context on language switching and lexical access in bilingual production. *Applied Psycholinguistics, 37*, 725–756. *https://doi.org/10.1017/S0142716415000223*	cued	2	**Spanish/English**	74.44	CTI 0 ms

49	Peeters, D. (2020). Bilingual switching between languages and listeners: Insights from immersive virtual reality. *Cognition, 195*, 104107. *https://doi.org/10.1016/j.cognition.2019.104107*	cued (contrast full switch/no switch)	0	**Dutch**/English	82.14	CTI 0 ms

50	Peeters, D., & Dijkstra, T. (2018). Sustained inhibition of the native language in bilingual language production: A virtual reality approach. *Bilingualism: Language and Cognition, 21*, 1035–1061. *https://doi.org/10.1017/S1366728917000396*	cued	4	**Dutch**/English	76.86 74.56 80.23 75.73	CTI 0 ms

51	Philipp, A. M., Gade, M., & Koch, I. (2007). Inhibitory processes in language switching: Evidence from switching language-defined response sets. *European Journal of Cognitive Psychology, 19*, 395–416. *https://doi.org/10.1080/09541440600758812*	cued	6	**German**/English/French	No self-rated proficiency scores	CTI 100/1000 ms

52	Prior, A., & Gollan, T. H. (2011). Good language-switchers are good task-switchers: Evidence from Spanish – English and Mandarin –English bilinguals. *Journal of the International Neuropsychological Society, 17*, 682–691. *https://doi.org/10.1017/S1355617711000580*	cued	2	**Spanish**/English **Chinese**/English	85.07 64.71	CTI 250 ms

53	Prior, A., & Gollan, T. H. (2013). The elusive link between language control and executive control: A case of limited transfer. *Journal of Cognitive Psychology, 25*, 622–645. *https://doi.org/10.1080/20445911.2013.821993*	cued	4	Spanish/**English** Chinese/**English** **Hebrew**/English **English**/various	42.03 82.86 67.16 90.77	CTI 250 ms

54	Reynolds, M. G., Schlöffel, S., & Peressotti, F. (2016). Asymmetric switch costs in numeral naming and number word reading: Implications for models of bilingual language production. *Frontiers in Psychology, 6*. *https://doi.org/10.3389/fpsyg.2015.02011*	reading aloud/cued/alternating runs	10	**Italian/**English **English**/French	50.14 50.14 No self-rated proficiency scores	not applicable

55	Santesteban, M., & Costa, A. (2016). Are cognate words “special”? In J. W. Schwieter (Hrsg.), Cognitive control and consequences of multilingualism (Bd. 2, S. 97–125). John Benjamins Publishing Company.	cued	4	**Spanish**/Catalan	95.72 54.14	CTI 2000 ms

56	Segal, D., Stasenko, A., & Gollan, T. H. (2019). More evidence that a switch is not (always) a switch: Binning bilinguals reveals dissociations between task and language switching. *Journal of Experimental Psychology: General, 148*, 501–519. *https://doi.org/10.1037/xge0000515*	cued	1	**English/**Spanish	90.91	CTI 250 ms

57	Slevc, L. R., Davey, N. S., & Linck, J. A. (2016). A new look at “the hard problem” of bilingual lexical access: Evidence for language-switch costs with univalent stimuli. *Journal of Cognitive Psychology, 28*, 385–395. *https://doi.org/10.1080/20445911.2016.1152274*	reading aloud	2	**Chinese/**English	73.43 73.43	not applicable

58	Stasenko, A., Matt, G. E., & Gollan, T. H. (2017). A relative bilingual advantage in switching with preparation: Nuanced explorations of the proposed association between bilingualism and task switching. *Journal of Experimental Psychology: General, 146*, 1527–1550. *https://psycnet.apa.org/doi/10.1037/xge0000340*	cued	8	**Spanish/**English	90.77 90.77 90.77 90.77 23.19 23.19 23.19 23.19	CTI 116/1016 ms

59	Timmer, K., Christoffels, I. K., & Costa, A. (2019). On the flexibility of bilingual language control: The effect of language context. Bilingualism: Language and Cognition, 22, 555–568. *https://doi.org/10.1017/S1366728918000329*	cued	2	**Dutch/**English	74.00 79.00	CTI 0 ms

60	Timmermeister, M., Leseman, P., Wijnen, F., & Blom, E. (2020). No Bilingual Benefits Despite Relations Between Language Switching and Task Switching. *Frontiers in Psychology*, 11. *https://doi.org/10.3389/fpsyg.2020.01832*	cued	0	**Dutch/**Turkish	114.23*	CTI 650 ms

61	Verhoef, K., Roelofs, A., & Chwilla, D. J. (2009). Role of inhibition in language switching: Evidence from event-related brain potentials in overt picture naming. *Cognition, 110*, 84–99. *https://doi.org/10.1016/j.cognition.2008.10.013*	cued	1	**Dutch**/English	51.40	CTI 500/1250 ms

62	Verhoef, K. M. W., Roelofs, A., & Chwilla, D. J. (2010). Electrophysiological evidence for endogenous control of attention in switching between languages in overt picture naming. *Journal of Cognitive Neuroscience*, 22, 1832–1843. *https://doi.org/10.1162/jocn.2009.21291*	cued	4	**Dutch/**English	55.00 55.00 55.00 55.00	CTI 500 ms

63	Vorwerg, C. C., Suntharam, S., & Morand, M.-A. (2019). Language control and lexical access in diglossic speech production: Evidence from variety switching in speakers of Swiss German. *Journal of Memory and Language, 107*, 40–53. *https://doi.org/10.1016/j.jml.2019.03.007*	cued	4	**Swiss German**/German **Swiss German**/Tamil	100.00 100.00 83.33 83.33	CTI 0 ms

64	Weissberger, G. H., Wierenga, C. E., Bondi, M. W., & Gollan, T. H. (2012). Partially overlapping mechanisms of language and task control in young and older bilinguals. *Psychology and Aging, 27*, 959–974. *https://doi.org/10.1037/a0028281*	cued	2	**English**/Spanish	95.38 98.31	CTI 2500 ms

65	Wong, W. L., & Maurer, U. (2021). The effects of input and output modalities on language switching between Chinese and English. *Bilingualism: Language and Cognition*, 1–11. *https://doi.org/10.1017/S136672892100002X*	alternating runs	2	**Chinese/**English	76.82	not applicable

66	Wu, J., Kang, C., Ma, F., Gao, X., & Guo, T. (2018). The influence of short-term language-switching training on the plasticity of the cognitive control mechanism in bilingual word production. *Quarterly Journal of Experimental Psychology, 71*, 2115–2128. *https://doi.org/10.1177/1747021817737520*	cued	2	**Chinese/**English	65.69 62.24	CTI 0 ms

67	Wu, J., Yang, J., Chen, M., Li, S., Zhang, Z., Kang, C., Ding, G., & Guo, T. (2019). Brain network reconfiguration for language and domain-general cognitive control in bilinguals. *NeuroImage, 199*, 454–465. *https://doi.org/10.1016/j.neuroimage.2019.06.022*	cued	0	**Chinese/**English	69.32	CTI 0 ms

68	Zhang, Y., Cao, N., Yue, C., Dai, L., & Wu, Y. J. (2020). The Interplay Between Language Form and Concept During Language Switching: A Behavioral Investigation. Frontiers in Psychology, 11, 791. *https://doi.org/10.3389/fpsyg.2020.00791*	cued	3	**Chinese/**English	65.83	CTI 0 ms

69	Zhang, M., Wang, X., Wang, F., & Liu, H. (2019). Effect of Cognitive Style on Language Control During Joint Language Switching: An ERP Study. *Journal of psycholinguistic research, 49*, 383–400. *https://doi.org/10.1007/s10936-019-09682-7*	cued	4	**Chinese/**English	65.38 60.00	CTI 750 ms

70	Zheng, X., Roelofs, A., Farquhar, J., & Lemhöfer, K. (2018). Monitoring of language selection errors in switching: Not all about conflict. *PLOS ONE, 13*, e0200397. *https://doi.org/10.1371/journal.pone.0200397*	cued	0	**Dutch**/English	86.00	CTI 0 ms

71	Zheng, X., Roelofs, A., Erkan, H., & Lemhöfer, K. (2020). Dynamics of inhibitory control during bilingual speech production: An electrophysiological study. *Neuropsychologia*, no pagination specified. *https://doi.org/10.1016/j.neuropsychologia.2020.107387*	cued	0	**Dutch**/English	84.00	CTI 0 ms

72	Zhu, J. D., Seymour, R. A., Szakay, A., & Sowman, P. F. (2020). Neuro-dynamics of executive control in bilingual language switching: An MEG study. *Cognition, 199*, 104247. *https://doi.org/10.1016/j.cognition.2020.104247*	cued	0	**Chinese/**English	60.00	CTI 750 ms

73	Zhu, J. D., & Sowman, P. F. (2020). Whole-Language and Item-Specific Inhibition in Bilingual Language Switching: The Role of Domain–General Inhibitory Control. *Brain Sciences, 10*, 517. *https://doi.org/10.3390/brainsci10080517*	cued	6	**Chinese/**English	87.68^†^ 77.96	CTI 0 ms


*Note*: ^1^ The dominant language (L1) is printed in bold. ^2^ Dominant/less dominant proficiency values refer to the values given in the respective study. When no dominant language proficiency values were reported, the highest possible values of the respective measurement for less dominant language proficiency assessment were used. When different conditions/samples were assessed, we decided – for the sake of transparency – to report all values, explaining why there are several less dominant/dominant proficiency values. CTI = Cue Target Interval, TCI = Target Cue Interval, RCI = Response Cue Interval *PPVT receptive vocabulary in Dutch and Turkish (the dominance ratio was clarified with the authors); ^†^ based on MINT Scores in L1 and L2; ^+^based on LexTaleScores. ^§^ SOA was manipulated between subjects only in Experiment 5; only descriptive data from short (500 ms) and long (800 ms) were requested; ^$^ only Experiment 1 considered as Experiment 2 used blocked sentence conditions; ^#^ only Experiment 1 taken into account as no switch-specific instructions were given for Experiment 2.

### Inclusion criteria

Given that we wanted to provide a comprehensive overview based on available data, we decided for broad inclusion criteria. That is, on the one hand we included all age groups as switch costs arise as a reliable effect across different age groups (i.e., children, young adults, and older adults), suggesting quantitative but not necessarily qualitative differences in underlying processes (Gollan et al., 2014; Gross & Kaushanskaya, 2015). In addition, we included different language-switching paradigms as language switch costs do not seem confined to certain paradigms, but their asymmetry might be (Declerck et al., 2013; Gollan & Ferreira, 2009; Meuter & Allport, 1999). Thus, we opted for a broad database and included potentially influential characteristics (e.g., the type of language-switching paradigm) in later auxiliary analyses. We considered published data sets using paradigms asking participants to switch among one or more language pairs differing in dominance (i.e., L1/L2 or L1/L2 plus L1/L3 and L2/L3, see e.g., Costa et al., 2006). We also included data from training studies (i.e., pre-test data when language switching was first assessed, Prior & Gollan, 2013; Wu et al., 2018). In case neuronal data were collected by means of electroencephalogram (EEG), functional magnetic resonance imaging (fMRI), magnet encephalogram (MEG), or transcranial magnetic stimulation (TMS), we used behavioural results only.

As dependent measures, we used language-specific vocal production RTs as this is the most used measure when assessing language switching. Moreover, these responses are target of the control processes proposed by models in language control (Declerck & Philipp, 2015). That is, we used mean RTs for each language in each language transition condition (switch vs. repetition) and language dominance assessment (dominant vs. less dominant language) obtained during stimulus naming, with a focus on single words as answers. The distinction between dominant and less dominant language was based on information given in the respective studies and indicated by the authors, mostly referring to the dominant language as L1 or first acquired language or in case of L2/L3 switching as L2 (see also ***Table 1*** in which the dominant language is printed in bold as inferred from the study). Please note that some studies used objective assessments of language dominance (Gullifer et al., 2013; Prior & Gollan, 2011, indexed in ***Table 1***), whereas others only report self-assessments (Gollan & Ferreira, 2009).

With regards to the type of paradigms to assess language switching, we included cued language switching (e.g., Meuter & Allport, 1999; Philipp et al., 2007), alternating-runs language switching (e.g., Declerck et al., 2013; Jylkkä et al., 2018) as well as voluntary language switching (e.g., Gollan & Ferreira, 2009; Gross & Kaushanskaya, 2015) and word reading studies (Macizo et al., 2012; Reynolds et al. 2016).

### Exclusion criteria

Applying the inclusion criteria mentioned above also led to the exclusion of some studies. First, by constraining to vocal response RTs of single words, we discarded (1) studies using eye movements (Byers-Heinlein et al., 2017; Goldrick et al., 2014; Potter et al., 2018); (2) studies examining responses with more than a single word (e.g., uttering a sentence, e.g., Declerck et al., 2017; Tarlowski et al., 2013); and (3) studies including comprehension tasks in which key press responses were mainly used and therefore lack the planning processes required for speaking (e.g., Macizo et al., 2012; Thomas & Allport, 2000; von Studnitz & Green, 1997, 2002).

Second, we excluded studies assessing switches among three languages within a block (Branzi et al., 2016; de Bruin et al., 2014). The reason is that the set-up of required processes when switching among three languages might differ in number and type of involved processes, that is, information accumulation processes before a word is uttered (Hick, 1952). Therefore, involved processes reflected in mean RT level might be altered when switching among three languages, making comparisons to studies involving only two languages difficult. Furthermore, most studies using asymmetrical switch costs as indication of underlying processes are usually concerned with the difference in dominance between two languages, having one more relatively dominant language in addition to a relatively weaker, less dominant language. Please note that this exclusion criterion also leads to the exclusion of all studies or experiments assessing so-called n-2 language repetition costs when participants switched among three languages within one block (e.g., Babcock & Vallesi, 2015; Guo et al., 2013; Philipp et al., 2007; Experiment 2; Philipp & Koch, 2009; see Koch et al., 2010, for a review). N-2 repetition costs are seen when analysing performance when switching among three languages in triplets for which the language in trial n (actual trial) is the same as the one two trials before (n-2 repetition, e.g., L1, L2, **L1**) compared to triplets in which the language in the actual trial is different from the language two trials before prior to that (n-2 switch, e.g., L3, L2, **L1**). N-2 language repetitions costs can be interpreted as an index of persisting inhibition when returning to a language from which one previously switched away. Yet, because there are only few studies investigating n-2 language repetition costs, we decided to leave them out in the present meta-analysis.

Third, all studies were excluded that assessed a language-switching paradigm embedded in other paradigms, such as the Psychological Refractory Period (PRP) paradigm (e.g., Hirsch et al., 2015) or joint language switching (e.g., Gambi & Hartsuiker, 2016). Fourth, we excluded studies that did not analyse language switch costs but focussed more on interference effects between languages (Costa et al., 1999; Emmorey et al., 2008; Fu et al., 2017; Gollan & Goldrick, 2016; Kohnert et al., 1999; Runnqvist et al., 2012; Schwieter & Sunderman, 2009). Fifth, to stay with one type of response modality, we excluded studies that assessed switching between two languages using different modalities for production (i.e., spoken and sign language; Dias et al., 2017; Kaufmann et al., 2018; Schaeffner et al., 2017). Finally, we discarded studies reporting no by-language analyses (Branzi et al., 2016; Declerck, Grainger, et al., 2017; Weissberger et al., 2015), studies constraining their analysis to the less dominant language only (Contreras-Saavedra et al., 2020), and those re-analysing published (and included) data (e.g., Fu et al., 2017).

## Data Analysis Strategy

To establish the necessity to account for *asymmetrical language switch costs* and the *reversed language dominance effect* when switching between languages, we analysed mean RTs for language switch and repetition conditions in either the dominant or less dominant language in a multilevel regression model (i.e., Newell & Dunn, 2008; Prince et al., 2012; see Rey-Mermet & Gade, 2018; Verhaeghen & De Meersman, 1998, for examples using this meta-analytical approach). This type of analysis was chosen to circumvent the problem of decomposing the interaction (i.e., the language transition by language dominance interaction being indicative of asymmetrical switch costs) into simple contrasts. This would have been necessary to identify the source of the significant interaction in a 2 × 2 within-subject design as well as to compute the effect sizes. These effects sizes would have formed the data basis of a conventional meta-analysis (see, e.g., Cumming, 2013; Field, 2013; Field & Gillett, 2010). However, although mostly inferential statistical values of the interaction term were reported in the published studies, the values for the simple contrast of the interaction were not. Furthermore, given that many studies assessed more than one experiment, using a multilevel regression approach also allowed us to model the different conditions assessed in a study as random effects and prevented effect size aggregation which would be required for an effect-size based meta-analysis that conventionally includes one effect size per study (Cumming, 2013).

To accomplish our analysis, we extracted mean RTs for switch and repetition conditions by language and by assessed experimental condition. Additional within-subject manipulations (i.e., timing manipulations such as cue-target intervals, different tasks such as picture or digit naming) or within-study manipulations (i.e., different samples and languages) were coded as random effects. Overall, this led to 239 random effect levels out of 73 studies that comprised 956 data points (239 × 4 including the factors dominance [dominant vs. less -dominant language] and transition [language switch vs. language repetition] with two levels each). No data trimming was applied but we did run an auxiliary analysis excluding extreme values. Next, the language dominance ratio in percent (less dominant/dominant language proficiency) was used as continuous variable in an auxiliary analysis to investigate the impact of language dominance on switch costs and the reversed language dominance effect.

When using inferential statistical approaches to compute the linear mixed effects model, no convergence was reached, and the models ran into singularities. As we found no theoretically or statistically sound way to reduce the random effects structure and still keep all information about the occurrence of the switch-cost asymmetry, we turned to a Bayesian approach (Kruschke, 2014). The Bayesian approach recently gained increasing support as it is more flexible and does not apply an arbitrary cut-off criterion (*p*-values; e.g., Wagenmakers et al., 2017). Furthermore, the Bayesian approach can deal with a relatively small number of data points even for more complex models. Given the ratio between number of studies and conditions assessed within this study (3.27, as 239 random effects were nested in 73 studies), our dataset benefited from using this approach (Sorensen et al., 2016).

Our model included the dominance factor, the language transition factor as well as the interaction between the two, which we assessed using the brms package (Bürkner, 2017). The full model thus reads:

\begin{array}{l}
R{T_{{\rm{ij}}}} = {\beta _0} + {\beta _1}\ language\ dominance + {\beta _2}\ language\ transition + {\beta _3}\ language\ dominance\ x\\
\quad\quad\quad language\ transition + ({b_{0i}} + {b_{1i}}{\kern 1pt} R{T_{{\rm{ij}}}} + {\varepsilon _{{\rm{ij}}}})
\end{array}

with *RT*_ij_ being the average response time for condition j in study i. Dominance was contrast-coded with –1 being assigned to the dominant language (as inferred from the study, or by contacting the authors) and 1 to the less dominant language. *β_1_* is the effect of language dominance (dominant language vs. less dominant language) on the intercept. In this coding, a negative point estimate along with a credible interval excluding zero would speak for better performance (shorter RTs) for the dominant language, whereas a positive point estimate (and a credible interval excluding zero) would be in favour of better performance in the less dominant language (i.e., the reverse language dominance effect). Language transition was also contrast-coded for language repetition (–1) and language switches (1). *β_2_* is the effect of language transition on the intercept. In this coding, switch costs would go along with a positive point estimate and a credible interval excluding zero. Finally, *β_3_* is the effect of language dominance on language transition. In this coding, a negative point estimate and a credible interval excluding zero provides supporting evidence for larger switch costs with the dominant language than the less dominant language (i.e., asymmetrical switch costs). *b*_0_*_i_* is the random intercept for study i, *b*_1_*_i_* is the random slope for study and *ɛ*_ij_ is the residual for condition j in study i, in case more than one condition was assessed within this study.

In addition to the interpretation of the credible interval, we also ran a model comparison that should identify the better fitting model to the present data. In this analysis, we compared the model including the interaction to a model that did not include the interaction (see Rey-Mermet & Gade, 2018, for a similar logic). In case the model with the interaction fits the data better, this would provide additional evidence for the asymmetry of language switch costs. To this end, we fit a second model without the interaction term and compared it to the model with the interaction term using the leave-one-out logic (Bürkner, 2017):

R{T_{{\rm{ij}}}} = {\beta _0} + {\beta _1}\ language\ dominance + {\beta _2}\ language\ transition + ({b_{0i}} + {b_{1i}}{\kern 1pt} R{T_{i{\rm{j}}}} + {\varepsilon _{{\rm{ij}}}})

All models were fit with 1000 iterations for warm-up and 5000 iterations for sampling using the brms package (Version 2.15.0, Bürkner, 2017) and R Version 3.63 (R Core Team, 2020). Priors were set as shown in ***Table 2*** and obtained from the get_prior function in brms. Those priors reflect the Student’s *t*-distribution with degrees of freedom, mean and standard deviation from which they were drawn.

**Table 2:** Priors for the fitted models using all data points.


*PARAMETER*	*PRIOR*

Intercept	t(3, 847.5, 215)

b	normal (0, 10)

sd	t(3, 0, 215)


Using priors from the Student’s *t*-distribution is recommended in Stan (underlying brms) in case data are presumed to be taken from a Gaussian distribution, as we did in the present study (Stan Development Team, 2018). The correlation among random effects (i.e., conditions in studies) was fit using the Cholesky factor to specify the correlation matrix (Bürkner, 2017). The b parameter was presumed to come from a normal distribution with mean = 0 and standard deviation = 10. Overall, these priors can be conceived as weakly informative.

## Results

**Figure 1:**
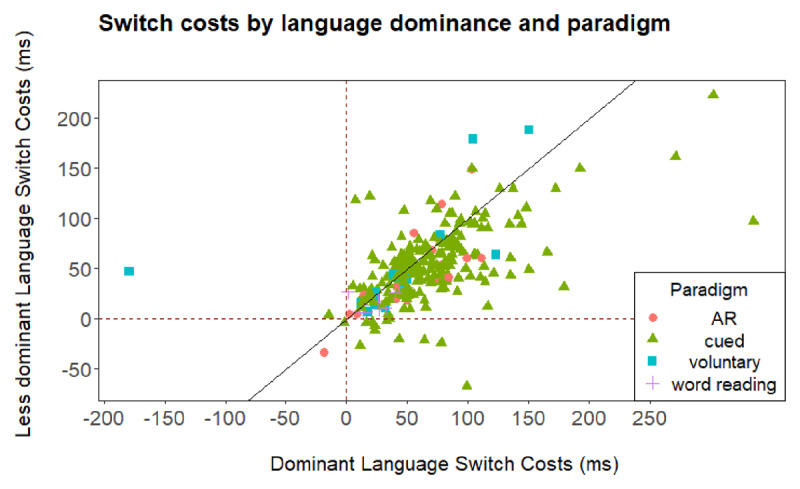
Language switch cost as a function of language dominance and paradigm for all data points included in the meta-analysis. AR stands for alternating runs.

**Figure 2:**
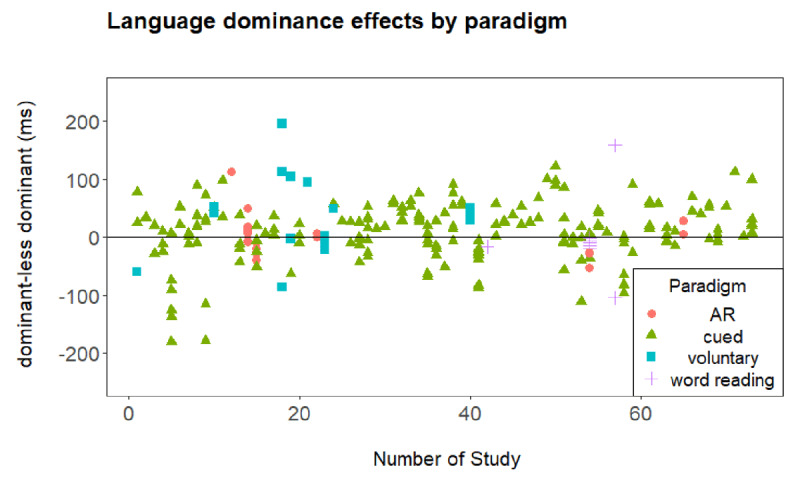
Language dominance effects (i.e., difference between mean RT in the dominant and less dominant language in mixed-language blocks) and paradigm for all data points included in the meta-analysis. Number of Study refers to the numbering of studies given in ***Table 1***. AR stands for alternating runs.

In our first analysis, we explored the possible interplay between the two effects plotted in ***Figure 1*** and ***2***, namely the asymmetrical switch costs and the reversed language dominance effect. More specifically, we report the correlation between both effects.

In a second step, we present the model including the interaction between dominance and transition in addition to the main effects of language dominance and language transition. Then, the model without the interaction will be presented and discussed. Finally, we will perform a model comparison using the leave-one out algorithm of the brms package (Bürkner, 2017; Sorensen et al., 2016).

Next to these main analyses, we performed auxiliary analyses to investigate conditions under which the effects are more likely to be observed. The first auxiliary analysis considers the language proficiency ratio, calculated as the reported proficiency in the less dominant language divided by that in the dominant language. The second auxiliary analysis focused on studies using cued language switching only, given that those studies made up most of the included data points (i.e., 192 of 239; 80%). In a third analysis, we used only those data (cued language switching with short preparation time) that have been suggested to be most likely to yield asymmetrical switch costs (Bobb & Wodniecka, 2013). Further auxiliary analyses excluding outliers, considering single-language block performance and paradigm types different from cued language switching can be found on OSF (*https://osf.io/ukjq4/*).

### Interplay of the reversed language dominance effect and asymmetrical switch costs?

To examine a potential interplay of the reversed language dominance effect and asymmetrical switch costs, we calculated a correlation between dominant language switch costs and difference in dominance between dominant and less dominant language. A negative correlation (i.e., larger dominant switch costs but still shorter RTs in the dominant language) could dissociate proactive and reactive inhibitory control. However, a positive correlation (i.e., larger dominant switch costs and longer RTs in the dominant language) would suggest a spill over from proactive to reactive language control. Yet, we observed a small negative correlation that was not significant, *r*(237) = –.12, *p* = .07. Thus, the data did not support contingent influences of proactive language control (indicated by the reversed language dominance effect) on reactive language control (indicated by asymmetrical switch costs).

### Full model with interaction

In ***Figure 1***, we plotted switch costs when switching to the dominant or less dominant language as a function of type of experimental paradigm. Data points below the diagonal reflect larger switch costs when switching to the dominant language, whereas data points above the diagonal reflect larger switch costs when switching to the less dominant language. The diagonal itself reflects equal costs when switching in the dominant or less dominant language, the origin (0|0) is marked with the dashed lines.

***Figure 2*** plots the mean RT difference between the dominant and less dominant language in mixed-language blocks being indicative of a language dominance effect in either direction. Slower performance in the dominant language (i.e., a positive difference and a reversed language dominance effect) is represented above the horizontal line, whereas slower performance in the less dominant language is presented below the line, again with reference to the different paradigms. The horizontal line at zero refers to no difference in RT for the dominant and less dominant language, respectively, and thus no dominance effect in either direction.

The full model converged without warning. The obtained parameter estimates, including their credible intervals and the number of effective sample size (ESS), can be found in ***Table 3***.

**Table 3:** Summary of model diagnostics and parameters estimated as well as credible intervals for the model including the interaction.


	ESTIMATE	ESTIMATED ERROR	LOWER 95%	UPPER 95%	Ȓ	BULK ESS	TAIL ESS

Intercept	895.25	23.15	850.11	941.67	1	556	1057

Language Dominance	–4.44	2.95	–10.3	1.40	1	12977	11174

Language Transition	27.13	2.85	21.58	32.68	1	14162	11666

Language Dominance * Language Transition	–3.0	2.88	–8.61	2.68	1	13661	11365


*Note*: Estimated mean of the posterior distributions, estimated error of the posterior distributions as well lower and upper 95% credible intervals of the posterior distributions, Ȓ as index for convergence, as well as effective sample size (ESS) for bulk and tail. Remember that language dominance and language transition were contrast-coded with –1 and 1 for dominant and repetition as for less dominant and switch.

The posterior distribution of our language dominance factor spans both positive and negative values and thus showed no evidence for the existence of a reversed language dominance effect, but also no evidence for a language dominance effect for the dominant language. The posterior distribution of the language transition factor was positive throughout, establishing a reliable presence of switch costs. The point estimate parameter for the interaction was negative, which indicates numerically larger switch costs for the dominant language, but the posterior distribution for the interaction (i.e., the 95% credible interval) contains also positive values, which suggests that the presence of the interaction is not supported by the present data.

However, a closer inspection of ***Figure 1*** suggests some outlying data points. We identified six outlying data points with switch costs being 3 standard deviations above or below the mean switch costs in either the dominant (M = 65.75 ms, SD = 47.89) or the less dominant language (M = 52.70 ms, SD = 37.83). Performing a similar analysis as the one presented above, but having removed these outliers, did not change the overall pattern of results.^1^ The results of this analysis can be found on OSF (*https://osf.io/ukjq4/*). Based on this outcome, we decided to focus on the complete data set for the following analyses.

We also computed a Bayes Version of R^2^ to assess model fit and found 81.1% variance explained on average by the model. Thus, the data were well accounted for by the model given that we entered all possible parameters and their combination. Nevertheless, there was a risk of overfitting (Bates et al., 2018), which means there is no variance left to explain, and most data are accounted for by means of model specification. To control for overfitting, we also fitted the more parsimonious model without the interaction.

### Model without the interaction

To further establish the support for the interaction between language dominance and language transition (i.e., asymmetrical switch costs), we computed a second model that did not include the interaction and assessed its fit. Priors for the model without the interaction were identical to the model with the interaction. The model converged without warnings, obtained parameter estimates can be found in ***Table 4***.

**Table 4:** Summary of model diagnostics and parameters estimated as well as credible intervals for the model without the interaction.


	ESTIMATE	ESTIMATED ERROR	LOWER 95%	UPPER 95%	Ȓ	BULK ESS	TAIL ESS

Intercept	892.04	23.64	844.95	937.02	1.01	368	864

Language Dominance	–4.42	2.92	–10.14	1.30	1	14498	11961

Language Transition	27.08	2.92	21.33	32.78	1	18111	11606


*Note*: Estimated mean of the posterior distribution, estimated error of the posterior distribution as well lower and upper 95%, credible intervals of the posterior distribution, Ȓ as index for convergence, as well as effective sample size (ESS) for bulk and tail. Remember that language dominance and language transition were contrast-coded with –1 and 1 for dominant and repetition as for less dominant and switch.

Mean Bayes-R^2^ was 81.1% for the model without the interaction, and this was numerically identical to the mean Bayes R^2^ of the model with the interaction, suggesting no difference in variance explanation between the two models. Parameter estimates did not change much for the intercept, the language transition, and the language dominance factor were comparable to the model that included the interaction. This suggests that the data were equally well captured by the model without the interaction as by the model with the interaction. Therefore, the last analysis was performed to decide which model provides a better fit of the data using the leave-one-out method (Vehtari et al., 2017).

### Model comparison

We compared the two models (with and without the interaction) using the leave–one-out method for Bayesian multilevel models (Bates et al., 2018; Bürkner, 2017; Sorensen et al., 2016; Vehtari et al., 2017). If the model without the interaction between language dominance and language transition would fit the data better, then this would suggest that the data are well described without the asymmetry in switch costs (see Rey-Mermet & Gade, 2018, for a similar approach).

When comparing the models with and without interaction (i.e., switch-cost asymmetry), the model without the interaction was shown to explain the data at least equally well as the model with the interaction. In fact, the difference between the two models was barely detectable. Additionally, there was not much of a difference between obtained fit diagnostics and estimates. Expected log posterior density difference was only –0.1 in favour of the model without the interaction, and model diagnostics and fit indices were highly similar (i.e., the widely applicable information criteria [WAIC, Watanabe, 2010] that were 11457.6 for the model with the interaction and 11457.3 for the model without the interaction). Thus, based on this model comparison, neither model outperforms the other in explaining the data. Hence, following the principle of parsimony as well as because the credible interval for the interaction term included zero, we conclude that no convincing evidence was found for the presence of the interaction and thus for asymmetrical switch costs.

In the following steps, we set out to investigate the variables that might influence the observation of asymmetrical switch costs and direction of the language dominance effect in auxiliary analyses. The main aim of these analyses was to establish conditions for which the credible interval for the interaction excluded zero, thus providing potentially clearer evidence for asymmetrical switch costs when switching between languages of different dominance.

### Auxiliary Analyses

In this section, we report a series of auxiliary analyses that might provide a deeper insight into potentially interesting factors that might modulate the observation of asymmetrical switch costs and language dominance effects. In a first auxiliary analysis, we considered language proficiency ratio as a potentially influential factor that could give rise to the observation of asymmetrical switch costs. Second, we considered paradigm-related effects given that asymmetrical switch costs might arise only with specific paradigms. Finally, we took suggestions for conditions yielding asymmetrical switch costs and tested them in a third auxiliary analysis.

#### Language proficiency ratio and asymmetrical switch costs

Across the 73 studies that were included in this meta-analysis, we encountered many different types of languages as well as participants that vary in proficiency ratio, ranging from completely balanced bilinguals (i.e., participants with a proficiency ratio equal to 100%, thus as proficient in the less dominant than in the dominant language, see ***Table 1***, fourth column) to less balanced bilinguals. Please note that it has been argued that highly proficient bilinguals do not necessary show asymmetrical switch costs (Costa & Santesteban, 2004). Therefore, participant’s proficiency ratio should affect the presence of an asymmetry in switch costs, and we included it as influential factor to account for the observation of switch cost asymmetries.

**Figure 3:**
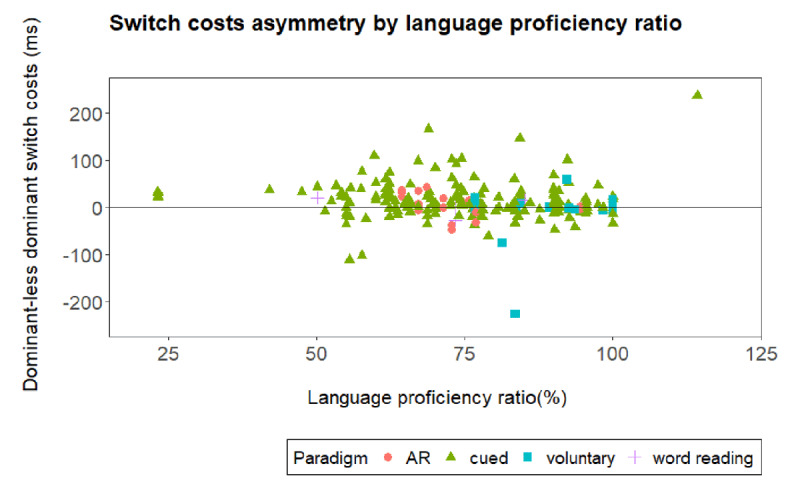
Asymmetrical switch costs (i.e., difference between switch costs for the dominant and less dominant language [switch costs dominant – switch costs less dominant]) by language proficiency ratio (%) and paradigm for all data points included providing language proficiency measures. AR stands for alternating runs.

In case no proficiency scores were given for either dominant and less dominant language, studies were excluded (80 datapoints, 6 studies, see ***Table 1***). In case more than one sample was assessed within a condition (i.e., Kleinmann & Gollan, 2016), we averaged across proficiency scores. We ran the same model with the interaction and now included proficiency ratio as an additional, continuous variable. ***Table 5*** provides the priors of the model fitted with proficiency as an additional variable and ***Table 6*** shows the obtained parameter estimates and their credible intervals. In case asymmetrical switch costs are constrained by the proficiency ratio, we would expect a three-way interaction of proficiency ratio, language transition, and language dominance. Please note that we had 107 different proficiency ratios (range 23.19 – 114.23%, mean = 75.56%, SD = 15.69%, see ***Table 1*** and ***Figure 3***) showing a rather diverse pattern across studies and thus results should be interpreted with caution as there is a risk of overfitting the model because of this large range of proficiency ratios.

**Table 5:** Priors for the fitted models with proficiency ratio as continuous variable.


*PARAMETER*	*PRIOR*

Intercept	t(3, 862, 212)

b	normal (0, 10)

sd	t(3, 0, 212)


**Table 6:** Summary of model diagnostics and parameters estimated as well as credible intervals for the model including the interaction for analysis with proficiency ratio as continuous variable.


	ESTIMATE	ESTIMATED ERROR	LOWER 95%	UPPER 95%	Ȓ	BULK ESS	TAIL ESS

Intercept	897.33	23.97	850.06	943.41	1.01	512	1116

Language Dominance	–0.08	5.96	–11.71	11.62	1	5975	8679

Language Transition	18.02	5.88	6.44	29.52	1	6554	10090

Proficiency Ratio	–9.77	9.54	–28.52	8.77	1	11794	11805

Language Dominance * Proficiency Ratio	–6.57	7.51	–21.33	8.08	1	5903	9044

Language Transition* Proficiency Ratio	11.48	7.42	–3.19	26.13	1	6325	9242

Language Dominance * Language Transition	–2.51	5.99	–14.17	9.26	1	6632	9747

Language Dominance * Language Transition * Proficiency Ratio	–0.69	7.52	–15.42	13.86	1	6717	9829


*Note*: Estimated mean of the posterior distributions, estimated error of the posterior distributions as well lower and upper 95% credible intervals of the posterior distributions, Ȓ as index for convergence, as well as effective sample size (ESS) for bulk and tail. Remember that language dominance and language transition were contrast-coded with –1 and 1 for dominant and repetition as for less dominant and switch, whereas Proficiency Ratio was obtained by dividing less dominant language proficiency rating by dominant language proficiency rating using the values and dominance assignments given in the study or by later queries, for scaling issues decimal values and not percent proficiency were used.

When inspecting the parameter estimates and their credible intervals in ***Table 6***, we found no evidence for a language dominance effect, as the posterior distribution for the language dominance factor contains both positive and negative values. As before we found evidence for switch costs as the posterior distribution for the language transition factor contains only positive values. Moreover, the interaction being indicative of an asymmetry in switch costs received no support, as the credible interval still included zero. So far, these results replicated those of our main analysis. Regarding the impact of language proficiency, the credible intervals for the language proficiency variable included zero. Thus, an influence of language proficiency on mean RT not supported, suggesting no overall benefit of more balanced proficiency. Furthermore, language proficiency ratio did not affect language transition (again the credible interval included zero). Finally, as the credible intervals for the three-way interaction included zero, there is no evidence that language proficiency ratio modulates the switch costs asymmetry (see ***Figure 3***).

To investigate whether the model including the language proficiency ratio fit the data better than the model including the asymmetry in the first place, we again performed model comparison. For model comparison, we refitted the reduced data set (excluding all studies not providing language proficiency values) with the interaction and no proficiency ratio and compared it to the model with the interaction and the language proficiency ratio. The model including the proficiency ratio fitted the data better (0.9 difference in ELPD) and thus should be preferred over the model not considering proficiency ratio between the less dominant and dominant language. Together, the present results show that although the results favour the model including the proficiency ratio, there was no support in this model for the three-way interaction between language dominance, language transition, and language proficiency ratio. Therefore, there is no evidence for a clear impact of language proficiency on asymmetrical switch costs probably because of the small amount of data included and the vast distribution of proficiency values.

#### Paradigm effects

As shown in ***Table 1***, our sample was heavily unbalanced regarding the paradigm types. Out of our 956 data points collected from 73 studies, 768 came from studies using cued language switching, 88 assessed alternating runs (sequences of AABB), 68 voluntary language switching, and 32 used a reading aloud task (see ***Table 1***).

In the second set of auxiliary analyses, we thus tested whether the diversity of the paradigms as well as their unbalance can explain our results. To this end, we focused on those studies that only used cued language switching as this paradigm was the one which was the most frequently used (see OSF for an analysis including all paradigm types with cued language switching as the reference). ***Table 7*** shows the priors for model fitting given cued language switching only.

**Table 7:** Priors for the fitted models using cued language switching only.


*PARAMETER*	*PRIOR*

Intercept	t(3, 877, 203.1)

b	normal (0, 10)

sd	t(3, 0, 203.1)


In ***Table 8***, we show the results obtained for fitting the model with the interaction based on data solely from cued language switching experiments. As before, we observed a small negative effect of language dominance whose credible interval included zero. Switch costs were reliably present. The interaction between language transition and language dominance suggested larger switch costs for the dominant language, but its credible interval included zero, which suggests that this interaction is not reliable. Thus, this pattern of results is similar to the pattern of results obtained in the main analysis.

**Table 8:** Summary of model diagnostics and parameters estimated as well as credible intervals for the model including the interaction for cued language switching only.


	ESTIMATE	ESTIMATED ERROR	LOWER 95%	UPPER 95%	Ȓ	BULK ESS	TAIL ESS

Intercept	891.16	22.05	847.92	935.33	1.01	615	1404

Language Dominance	–3.83	2.95	–9.68	1.97	1	17236	11852

Language Transition	28.55	2.95	22.7	34.33	1	17123	11503

Language Dominance * Language Transition	–3.78	2.96	–9.52	2.11	1	16434	10385


*Note*: Estimated mean of the posterior distributions, estimated error of the posterior distributions as well lower and upper 95% credible intervals of the posterior distributions, Ȓ as index for convergence, as well as effective sample size (ESS) for bulk and tail. Remember that language dominance and language transition were contrast-coded with –1 and 1 for dominant and repetition as for less dominant and switch.

Thus, there was no clear evidence for the presence of asymmetrical switch costs even when constraining the data to cued language switching only. However, one prominent design feature of the cued language switching paradigm is the use of different timing intervals between the presentation of the cue and the imperative stimulus, the cue-target interval (Meiran, 1996, 2014). It is commonly assumed that this interval is used to reconfigure the cognitive system in case of language switch (Bobb & Wodniecka, 2013). This opportunity to prepare might abolish asymmetrical switch costs (e.g., Verhoef et al., 2009). In a final auxiliary analysis, we therefore constrained the analysis within the cued language switching studies further and took only those studies in consideration that included no opportunity to prepare (i.e., a CTI of 0 or less than 120 ms, see ***Table 1***). This filtering leads to a final sample of 312 data points. Priors for the model with interaction can be found in ***Table 9*** and obtained parameter estimates in ***Table 10***.

**Table 9:** Priors for the fitted models using cued language switching with short CTI only.


*PARAMETER*	*PRIOR*

Intercept	t(3, 923, 170.5)

b	normal (0, 10)

sd	t(3, 0, 170.5)


**Table 10:** Summary of model diagnostics and parameters estimated as well as credible intervals for the model including the interaction for cued language switching with short CTI only.


	ESTIMATE	1 ERROR	LOWER 95%	UPPER 95%	Ȓ	BULK ESS	TAIL ESS

Intercept	927.93	32.84	863.1	992.17	1	1090	1998

Language Dominance	–8.83	3.6	–15.96	–1.79	1	10809	10835

Language Transition	29.64	3.64	22.4	36.79	1	9431	10531

Language Dominance * Language Transition	–2.94	3.56	–9.83	4.07	1	9949	10825


*Note*: Estimated mean of the posterior distributions, estimated error of the posterior distributions as well lower and upper 95% credible intervals of the posterior distributions, Ȓ as index for convergence, as well as effective sample size (ESS) for bulk and tail. Remember that language dominance and language transition were contrast-coded with –1 and 1 for dominant and repetition as for less dominant and switch.

***Table 10*** shows the results when fitting the model including the interaction between language dominance and language transition for data points obtained in studies using cued language switching only with no or short preparation time. Even though it has been proposed (Bobb & Wodniecka, 2013) that those conditions might be favourable for observing the interaction between language transition and language dominance (i.e., asymmetrical switch costs), the credible interval for the interaction still included zero. Again, switch costs were present and language dominance effects were absent.^2^

## General Discussion

We investigated the empirical support for two phenomena of high theoretical interest that are currently debated among researchers in the field of language control, namely the observation of asymmetrical switch costs when switching between languages of different dominance and a reversed language dominance effect in mixed-language blocks. Both measures are usually taken to reflect different forms of inhibitory language control. Asymmetrical switch costs, which is reflected in the interaction of language transition with language dominance, is typically taken as an index of reactive, transient inhibitory control (Bobb & Wodniecka, 2013; Declerck & Philipp, 2015). The reversed language dominance effect as shown by a main effect of dominance only, on the other hand, is taken as an index of proactive, sustained inhibitory control (Declerck, 2020; Declerck et al., 2020: Kleinman & Gollan, 2016).

A quantitative analysis, as the one performed in this study, extends narrative reviews in that it establishes the consistency of effects across a range of studies and thus assesses quantitatively whether and under what conditions those two effects are observed. The aim of this study was thus to establish whether either one of the empirical effects or even both effects – asymmetrical switch costs and the reversed language dominance effect – can inform the field about theoretical constraints in bilingual language processing models and to what degree the presence of one effect constrains the other. To this end, we collected and analysed published studies addressing both effects. In a first step we assessed the relationship between both effects by means of correlational analysis and observed no significant relationship. We then fitted a Bayesian Hierarchical regression model to be able to consider most published results modelling the standard 2 × 2 within-subject design with language dominance and language transition as fixed factors. Given our choice for a Bayesian approach and a hierarchical regression model, we could include all studies of interest and did not have to restrict the analyses. In addition, the auxiliary analyses provided a more fine-grained picture.

### Asymmetrical language switch costs

To investigate the evidence for asymmetrical language switch costs, our analysis was based on mean RTs as a function of language dominance and language transition. To establish the presence of an asymmetry, we examined parameters for both factors plus their interaction and focused on the parameter value of the interaction term that would indicate the presence of an asymmetry. The results show that the credible interval of the interaction parameter was broad and included zero, thus providing no empirical support for the presence of the interaction consistently in the present data.

Additionally, we compared this model to a model excluding the interaction. The difference in fit between the two models was barely existent. We then set out to identify possible factors that might constrain the observation of the interaction in the data published so far. Balanced bilinguals do not necessarily show asymmetrical switch cost but might solve language selection without reliance on transient inhibition (e.g., Costa et al., 2006; Costa & Santesteban, 2004; Schwieter & Sunderman, 2009). In our first auxiliary analysis, we added a language proficiency ratio measure as a continuous variable as proxy for the degree of balance. The ratio was obtained by dividing (self-assessed) language proficiency in the less dominant language by (self-assessed) language proficiency in the dominant language. Although this model fit the data better than the model without language proficiency ratio, no empirical support for the interaction could be detected in this analysis as well. We would like to mention that most data points of language proficiency came from self-assessment, which might be by itself problematic (Tomoschuk et al., 2019). Furthermore, we observed a broad range of different levels of proficiency ratio, which constrains the statistical model (see ***Figure 3***) in that it is likely to be overfitted given too few data points within each level of proficiency. However, in line with our findings, a recent study based on more than 400 participants also showed that asymmetrical switch costs were not affected by language balancing (Declerck et al., 2020), when it was measured with an objective language proficiency task (Gollan et al., 2012). One reason for these findings might be that bilingualism (and thus language dominance) is not a stable trait within a person. For example, even highly proficient bilinguals generally use one language more often than the other, depending on current demands and life stage (Marian & Hayakawa, 2020). Thus, there is reason to believe that the emergence and observation of asymmetrical switch costs might differ among and even within bilinguals, dependent on current demands. In line with the idea of contextually changing language proficiency and inferred bilingualism are studies that did not show asymmetrical switch costs in bilinguals with different language proficiency (for partial evidence, see also Bonfieni et al., 2019).

In line with this reasoning, the dominance patterns across languages have been proposed to be affected by even more local variables such as block length and context (i.e., training or immersion), reflecting the highly dynamic nature of language dominance and suggesting differential influences dependent on time scale (Wodniecka et al., 2020).

Given the prominence of asymmetrical switch costs in terms of the interaction between language dominance and language transition in the research literature (e.g., Meuter & Allport, 1999; Philipp et al., 2007), this finding might seem surprising, as asymmetrical switch costs are often assumed to be a replicable and robust effect. However, the present meta-analytical findings are in line with prior narrative reviews, which already questioned the reliability of this effect (Bobb & Wodniecka, 2013; Declerck & Philipp, 2015). Therefore, in a second auxiliary analysis, we only considered the most common paradigm under which asymmetrical switch costs have been observed, the cuing paradigm and still were left with no support for the interaction. In an even more fine-grained analysis, we used only the condition (i.e., no or short preparation time) for which it has been proposed that participants could not counteract the reactive inhibitory mechanism suppressing the more dominant language and in consequence give rise to the asymmetry (i.e., larger switch costs when switching to the more dominant language). In this further reduced data set, still no evidence for the interaction was found, even when proficiency was also considered as a factor.

One might argue that the restriction to only include published studies, next to the missing responses of authors on our requests for sharing data, might have limited our analysis and biased its outcome. Still, we collected data from 73 studies and opted for an analysis including all available data points. Thus, we are confident that the present meta-analysis provides a relatively comprehensive collection of available data.

### Language dominance effect

As regards language dominance, we found no overall indication for a reversed language dominance effect. Yet, we also found no empirical support for better performance of the dominant language relative to the less dominant language. When we went back to the studies that were included in this meta-analysis, we found a roughly equal distribution of studies for either one of the language-dominance patterns, suggesting a rather balanced sample (see ***Figure 2***), which explains the small parameter estimates obtained for the language dominance factor, lacking clear directionality. This impression was further confirmed by our auxiliary analyses that considered only those studies that assessed single language performance. In these analyses, which are reported on OSF (see *https://osf.io/ukjq4/*), we found that there was again no clear evidence of language dominance in mixed language blocks, relative to the language dominance in single language blocks, in either direction but a rather broad distribution of effects. Though, these analyses must be treated with caution because the number of data points was relatively small. That is, only 24 studies included single language blocks, and of these 24 studies, only 19 were analysed because only those included all relevant values.

In this context, it should be noted that there is some discussion about influencing factors for the direction of the language dominance effects, such as the type of paradigm that might elicit such a pattern, the effect of block length (Kleinman & Gollan, 2018), how balanced the bilinguals are (Declerck et al., 2020), and whether their current situation enforces active bilingualism (Anderson et al., 2020; Marian & Hayakawa, 2020; Wodniecka et al., 2020). So, further careful experimentation is needed to isolate factors that might influence the directionality of the effect. However, the currently available data suggest no clear direction of language dominance.

### Implications for inhibitory control in language processing

Both effects under consideration in the present work have been taken as indicators for inhibitory control, as proposed by the influential inhibitory control model by Green (1998). Yet, further theoretical refinement has dissected the rather broad notion of inhibition and linked it to different levels of selection. It has been assumed that there is a more global inhibition acting on the lexical level (i.e., making all particles of a language less accessible), which is linked to the notion of reversed language dominance (Green, 1998, but see also Casado et al., 2021). More in line with recent suggestions (Casado et al., 2021), our findings do not reveal consistent evidence for the reversed language dominance effects as support for the notion of global lexical level inhibition probably because of a missing assessment of current language dominance.

However, as for global inhibition, there are also other measures that can be used to examine more global inhibition, such as the blocked language order effect (e.g., Branzi et al., 2014; Degani et al., 2020; Kreiner & Degani, 2015; Misra et al., 2012; Van Assche et al., 2013). Therefore, the present finding of an overall lack of support for a reversed language dominance effect does not invalidate the inhibitory control framework but instead suggests that these empirical phenomena still lack a coherent explanation.

The same holds true for asymmetrical switch costs, which has been assumed to be a measure for reactive inhibition. It is notable that other experimental approaches provide consistent evidence for more specific, reactive inhibition in the sequential control of language processing (Philipp et al., 2007; Philipp & Koch, 2009) beyond asymmetrical switch costs (e.g., Branzi et al., 2016; Declerck et al., 2018, 2019; Eben & Declerck, 2019; Philipp et al., 2007). For instance, with a paradigm assessing switching among more than two languages, more convincing evidence for the role of inhibition has been observed in language switching studies (Babcock & Vallesi, 2015; Branzi et al., 2016; Declerck, Thoma, et al., 2015; Declerck & Philipp, 2018; Guo et al., 2013; Philipp et al., 2007; Philipp & Koch, 2009). In these studies, participants switched among three languages and so-called n-2 language repetition costs were observed when comparing the last trial of sequences like L1 – L2 – **L1** to language sequences of the type L3 – L2 – **L1**, suggesting persisting inhibition of a previously abandoned language (i.e., when returning to a language that was switched away two trials ago). These n-2 language repetition costs can be directly linked to commonly observed n-2 task repetition costs that are usually taken as markers for inhibition of competing task-sets (Gade et al., 2014; Kiesel et al., 2010; Koch et al., 2010, 2018; Mayr & Keele, 2000).

Hence, the present approach does not necessarily raise major questions about the role of inhibition in bilingual language selection. Instead, it suggests that the switch cost asymmetry and the direction of the overall language dominance effects simply should not be taken to provide strong support for any specific theoretical model because these effects lack empirical robustness and thus defy a coherent explanation. However, for inhibitory processes in the selection of languages, although improved and methodologically sound markers for inhibition have been around, the practice of assessing asymmetrical switch costs as a measure of inhibition as a necessary mechanism in the process of language selection is still widespread (for discussions see Declerck, Thoma, et al., 2015; Philipp et al., 2007). Yet, based on our findings we advocate that the reversed language dominance effect and asymmetrical switch costs should be treated more cautiously and more as an experimental effect that still requires further empirical work to uncover the underlying processes, which may or may not require inhibitory mechanisms.

## Conclusion

In our study, we aimed at assessing the evidence of two empirical markers of inhibitory processes in language control, namely asymmetrical switch costs as an index of reactive language control and the reversed language dominance effect as an index of proactive language control. The available data suggest that neither one nor the other is an empirically robust finding that will be consistently observed when switching between languages. Thus, these findings may have, at least currently, less theoretical implications than previously assumed. Further research specifying conditions under which those effects arise and employing objective measurement tools to assess current language dominance and the mechanisms that bring them about might change the present conclusions. This work will also inform about the relationship of the two forms of language control (i.e., proactive, sustained and reactive, transient) that are assumed to underly those effects. For the time being, the conditions for observing asymmetrical language switch costs and the reversed language dominance effect remain to be unravelled.

## Data Accessibility Statement

Raw data and analyses files can be found at OSF: *https://osf.io/ukjq4/*

## Footnotes

To foreshadow the next step, in comparison to the main analysis, the analyses excluding the outliers showed that the model comparison favoured the model without the interaction a bit more over the model with the interaction when using the leave-one-item out method (ELPD –0.2, WAIC –0.3, for model parameters and estimates see OSF: *https://osf.io/ukjq4/*).

Please note that we repeated this analysis also with proficiency ratio and similar results were obtained. We found no influence of language proficiency ratio at all or on asymmetrical switch costs.
