# Correction: Assessing the Evidence for Asymmetrical Switch Costs and Reversed Language Dominance Effects – A Meta-Analysis

**DOI:** 10.5334/joc.195

**Published:** 2021-11-10

**Authors:** Miriam Gade, Mathieu Declerck, Andrea M. Philipp, Alodie Rey-Mermet, Iring Koch

**Affiliations:** 1 Catholic University of Eichstätt-Ingolstadt, Department of Psychology, General Psychology; Medical School Berlin, Department of Sciences, Berlin, DE; 2 Vrije Universiteit Brussel, Department of Linguistics and Literary Studies, Brussels, BE; 3 RWTH Aachen University, Department of Psychology, Aachen, DE; 4 UniDistance Suisse, Brig, CH

**Keywords:** bilingualism, language control, cognitive control, language production

## Abstract

This article details a correction to: Gade, M., Declerck, M., Philipp, A. M., Rey-Mermet, A., & Koch, I. (2021). Assessing the Evidence for Asymmetrical Switch Costs and Reversed Language Dominance Effects – A Meta-Analysis. *Journal of Cognition, 4*(1), 55. DOI: *http://doi.org/10.5334/joc.186*

## Correction

In our publication “Assessing the Evidence for Asymmetrical Switch Costs and Reversed Language Dominance Effects – A Meta-Analysis.” Table 10 reports estimates for the auxiliary analysis on cued language switching with short CTI. In the accompanying text of this table, we state that no language dominance effect was observed. However, this statement is wrong, because we did in fact observe a reversed language dominance effect as indicated by the estimates. Yet, this effect was only present in this analysis but in no other (main) analyses. This error was caused by an oversight and identified by an attentive reader. Follow-up analyses of this observation can be found in the auxiliary materials published on OSF *https://osf.io/zjyqk/*.
